# Ways to Achieve Healthy and Sustainable Diets

**DOI:** 10.3390/nu18091372

**Published:** 2026-04-27

**Authors:** Verônica Cortez Ginani, Renata Puppin Zandonadi

**Affiliations:** Department of Nutrition, Faculty of Health Science, University of Brasília, P.O. Box 4569, Brasília 70904-970, DF, Brazil

## 1. Introduction

The relationship between food and sustainability has gained increasing visibility, particularly considering environmental management, the scarcity of natural resources, and the persistence of global undernourishment [1,2,3]. The demand for healthy, sustainable diets has emerged as a critical global priority, driven by the dual challenges of rising non-communicable diseases and the environmental impact of food systems [4,5]. As the relationship between food production, consumption, and planetary health becomes increasingly visible, there is an urgent need to transition toward dietary patterns that are not only nutritionally adequate but also environmentally respectful, culturally acceptable, and economically accessible [6].

Food choices, which reflect consumption and production methods, directly impact sustainability and quality of life, requiring a collective approach. Access to sufficient, nutritious, safe, and culturally protected food from sustainable production systems is a fundamental human right [3,7,8]. To realize this right and promote safe and accessible diets, research must be strategically directed towards public policies and governmental or private actions. Collective awareness of healthy food choices, coupled with nutritional education and consideration of external influences such as the media, is essential. Collaboration among the various actors involved in the formation of individuals aware of and committed to healthy eating will strengthen the guarantee of the right to food.

In this sense, the research topic “Ways to Achieve Healthy and Sustainable Diets” was conceived to summarize state-of-the-art research and practices that promote collective awareness and practical solutions for this transition, focusing on solutions to protect life, health, and the planet, especially for vulnerable populations. The contributions published herein reflect the multidisciplinary nature of this field, spanning food safety in school feeding programs to the psychological determinants of meat consumption and the role of digital technology in personalized nutrition.

## 2. Recent Developments and Knowledge Gaps

Recent years have seen expressive advancements in our understanding of sustainable food systems. The integration of the One Health approach has highlighted the indissociable link between human nutrition, animal health, and environmental integrity [9,10]. Although some authors have discussed the structural and conceptual issues of the One Health approach in depth, the topic remains urgent globally. Researchers point out aspects such as the lack of integration among actions and the emphasis on technical issues, which reduce critical approaches (social epidemiology, social sciences, political ecology). They highlight the need to broaden the discussion for better adaptation and functionality [11,12].

Furthermore, the development of plant-based alternatives and the exploration of bioactive compounds in traditional beverages like kombucha and tea have expanded the options for sustainable dietary choices [13,14].

However, several knowledge gaps remain. While the theoretical benefits of sustainable diets are well established, there is a lack of evidence on how to effectively implement these changes across diverse socio-economic contexts, particularly among vulnerable populations such as older adults or low-income families [15]. Moreover, the role of behavioral determinants requires further investigation to design more effective public health interventions. This research topic addresses these gaps by providing evidence on (i) the implementation of good agricultural practices in small-scale farming for government programs; (ii) the psychological and environmental factors influencing the intention to reduce meat consumption among young adults; (iii) the effectiveness of personalized dietary interventions using mobile technology in real-world scenarios; (iv) the nutritional adequacy of dietary patterns, including vegan, vegetarian, and omnivorous diets, in different cultural settings.

## 3. Conclusions and Future Directions

This collection of studies, devoted to summarizing state-of-the-art research and practices for achieving healthy and sustainable diets, encompasses a diverse range of studies, enlightening the field’s breadth. This is also portrayed in the different methods used in the studies. To further stimulate interest and engagement in this field, future research should focus on the following key areas: (i) scaling interventions, showing how to scale successful local interventions (e.g., school feeding programs) to national levels while maintaining food safety and sustainability standards; (ii) technological integration, exploring the potential of artificial intelligence and apps to provide real-time, context-specific guidance for sustainable food choices; (iii) life-course approaches, investigating how dietary needs and sustainable practices evolve from childhood through old age, with a focus on designing food solutions tailored for seniors; and (iv) policy and environment, analyzing the impact of workplace environments and corporate social responsibility on employee nutritional education and health promotion. By addressing these areas, the scientific community can provide policymakers, practitioners, and consumers with the tools needed to achieve a truly sustainable and healthy food future.
